# Effects of evolocumab on plasma coenzyme Q10 in patients with heterozygous familial hypercholesterolemia

**DOI:** 10.1016/j.athplu.2026.100575

**Published:** 2026-06-19

**Authors:** Hayato Tada, Yasuaki Takeji, Masayuki Takamura

**Affiliations:** Department of Cardiovascular Medicine, Kanazawa University Graduate School of Medical Sciences, Kanazawa, Japan

**Keywords:** Familial hypercholesterolemia, CoQ10, LDL cholesterol, Statin, Lp(a)

## Abstract

**Background and aims:**

Muscle symptoms are common with statin therapy, and reduced plasma coenzyme Q10 (CoQ10) has been proposed as a potential mechanism. Proprotein convertase subtilisin/kexin type 9 (PCSK9) inhibitors are widely used in patients with statin intolerance or severe hypercholesterolemia, including familial hypercholesterolemia (FH), but their effects on plasma CoQ10 remain unclear.

**Methods:**

We evaluated serum lipid profiles and plasma CoQ10 baseline and 4 weeks after adding evolocumab in patients with heterozygous FH receiving rosuvastatin 10−20 mg/day and ezetimibe 10 mg/day (N = 10; mean age, 52 years; 6 men). Plasma CoQ10 concentrations were determined by high-performance liquid chromatography.

**Results:**

Evolocumab markedly reduced LDL cholesterol and lipoprotein(a) [Lp(a)] (121 mg/dL to 64 mg/dL, −50.0%, p < 0.001; 51.8 nmol/L to 39.1 nmol/L, −23.6%, p = 0.002), whereas median CoQ10 levels were unchanged (1028.5 nmol/L to 1002.5 nmol/L, −0.2%, p = 0.33). Changes in CoQ10 did not correlate with reductions in LDL cholesterol or Lp(a).

**Conclusions:**

These findings indicate that evolocumab lowers atherogenic lipoproteins without reducing plasma CoQ10, suggesting a potential advantage for patients susceptible to statin-associated muscle symptoms.

## Highlights

• Evolocumab substantially reduced LDL cholesterol and Lp(a) levels in patients with HeFH.
• Evolocumab had no effect on plasma CoQ10 level in patients with HeFH.
• Changes of plasma CoQ10 by evolocumab was not correlated with changes of lipids.

## Graphical abstract

**Figure:**
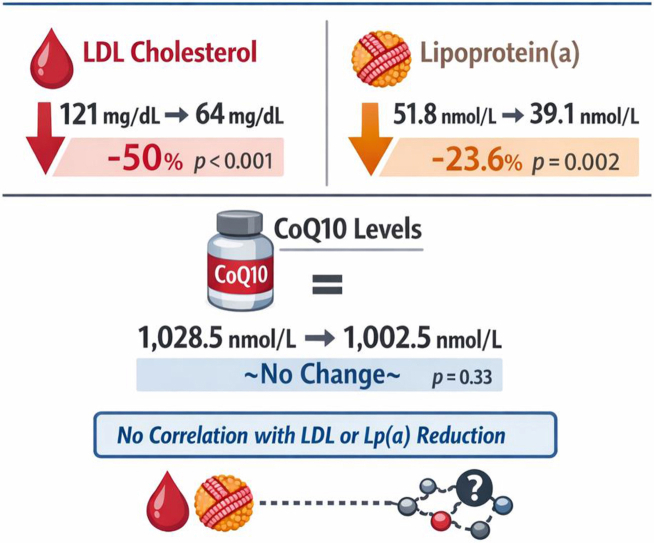

## 1 Introduction

Statins not only reduce LDL cholesterol levels but have also been shown in numerous clinical trials to suppress the progression of coronary artery disease, establishing them as cornerstone agents in the prevention of atherosclerotic disorders. Nevertheless, a proportion of patients develop muscle-related symptoms during statin therapy, necessitating alternative management strategies for statin intolerance [1]. Proprotein convertase subtilisin/kexin type 9 (PCSK9) inhibitors are widely used in this context, and their efficacy and safety profiles have been well documented [2,3]. Although PCSK9 inhibitors achieve greater reductions in LDL cholesterol than statins, they can be administered safely to patients who are unable to tolerate statins, indicating that statin intolerance is not attributable to LDL cholesterol lowering per se. Several mechanisms have been proposed to explain statin intolerance and statin-associated muscle symptoms [4,5]. One hypothesis is that statins inhibit hepatic cholesterol biosynthesis and, in doing so, also reduce circulating levels of coenzyme Q10 (CoQ10), which is synthesized through the same metabolic pathway. This reduction in CoQ10 has been suggested as a potential contributor to statin-induced muscle symptoms [6]. However, no data have been available regarding the effects of PCSK9 inhibitors on circulating CoQ10 levels. Therefore, we investigated serum lipid profiles and plasma CoQ10 concentrations before and after the addition of evolocumab in patients with heterozygous familial hypercholesterolemia (HeFH) who were receiving rosuvastatin and ezetimibe, and examined the associations between CoQ10 changes and reductions in LDL cholesterol and lipoprotein(a) [Lp(a)].

## 2 Materials and methods

### 2.1 Study population

We enrolled 10 patients with genetically confirmed heterozygous familial hypercholesterolemia (HeFH) who were admitted to Kanazawa University Hospital and initiated on evolocumab 140 mg every two weeks. All patients had been receiving rosuvastatin (10–20 mg/day) and ezetimibe (10 mg/day) prior to the introduction of evolocumab.

### 2.2 Clinical data collection

Serum lipid parameters—including LDL cholesterol, HDL cholesterol, lipoprotein(a) [Lp(a)], and plasma CoQ10 (ubiquinol-10 and ubiquinone-10)—were measured at baseline and 4 weeks after starting evolocumab (140 mg biweekly). LDL cholesterol, HDL cholesterol, and Lp(a) were quantified using enzymatic methods. Because Lp(a) was measured in mg/dL, values were converted to nmol/L using a standardized formula described previously [7]. Plasma ubiquinol-10 and ubiquinone-10 concentrations were determined by high-performance liquid chromatography (HPLC) [8]. The detection limit of plasma ubiquinol-10 and ubiquinone-10 is about 4 nmol/L with excellent reproducibilities, whose coefficient of variation were 3 to 4 %.

### 2.3 Ethical considerations

The study protocol was approved by the Ethics Committee of Kanazawa University. All procedures adhered to institutional and national ethical standards for human research and complied with the Declaration of Helsinki (1975, revised 2008). Written informed consent was obtained from all participants.

### 2.4 Statistical analysis

Median values were compared using the nonparametric Wilcoxon signed-rank test. Pearson's correlation coefficients were calculated to assess relationships among LDL cholesterol, Lp(a), and CoQ10. Statistical analyses were performed using R software (https://www.r-project.org). A p-value <0.05 was considered statistically significant.

## 3 Results

### 3.1 Clinical characteristics

The clinical characteristics of the study population are summarized in Table 1. The mean age of the patients was 52 years. All participants were receiving rosuvastatin (10–20 mg/day) and ezetimibe (10 mg/day). Under this treatment regimen, the median LDL cholesterol level was 121 mg/dL, the median Lp(a) level was 52.8 nmol/L, and the median baseline plasma CoQ10 (ubiquinol-10 plus ubiquinone-10) concentration was 1027.5 nmol/L.

**Table 1:** Baseline characteristics.

ID	Age (years)	Sex	LDL cholesterol (mg/dL)	Lp(a) (nmol/L)	CoQ10 (nmol/L)	HDL cholesterol (mg/dL)	Hypertension	Diabetes	Smoking	CAD	Medications
A	66	M	102	207.4	894	42	Yes	No	Yes	Yes	R20mg/E10mg
B	54	F	126	27.4	1234	55	No	No	No	No	R20mg/E10mg
C	68	F	98	5.6	945	43	Yes	No	No	Yes	R20mg/E10mg
D	34	M	134	10.4	1125	49	No	No	No	No	R20mg/E10mg
E	50	F	123	55.7	1044	47	Yes	No	No	No	R20mg/E10mg
F	24	M	145	104.5	1298	43	Yes	No	No	No	R10mg/E10mg
G	71	M	98	78.2	987	36	Yes	Yes	Yes	Yes	R20mg/E10mg
H	49	F	119	23.4	1067	54	No	No	No	No	R20mg/E10mg
I	44	M	128	116.7	1011	51	No	No	Yes	No	R20mg/E10mg
J	56	M	110	47.8	976	39	Yes	No	No	No	R20mg/E10mg
Summary	52 ± 15	male = 6 (60 %)	121 [104−128]	51.8 [24.4−98]	1027.5 [978.8−1110.5]	45 [42−51]	6 (60 %)	1 (10 %)	3 (30 %)	3 (30 %)	NA

Lp(a): lipoprotein(a), CoQ10: Coenzyme Q10, CAD: coronary artery disease, M: male, F: female, R: rosuvastatin, E: ezetimibe, NA: not applicable.

### 3.2 Changes in lipid parameters and CoQ10 with evolocumab

**Fig. 1:**
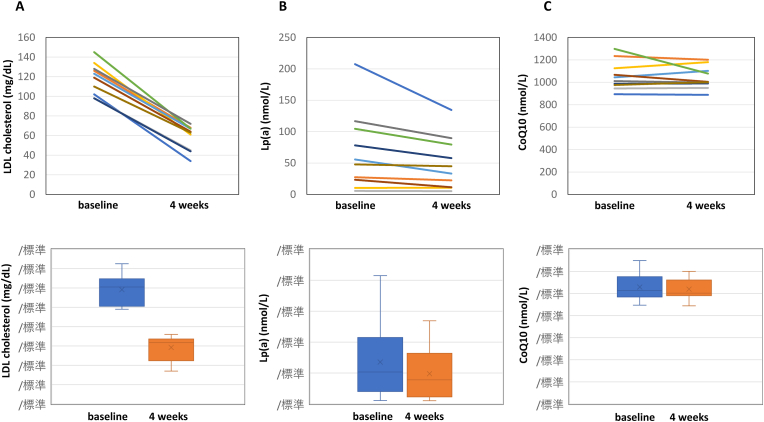
Changes of serum lipids and CoQ10 by evolocumab **A. LDL cholesterol:** The upper panel shows the line graph, and the lower panel shows the boxplot (baseline: blue, 4 weeks: orange). **B. Lp(a):** The upper panel shows the line graph, and the lower panel shows the boxplot (baseline: blue, 4 weeks: orange). **C. CoQ10:** The upper panel shows the line graph, and the lower panel shows the boxplot (baseline: blue, 4 weeks: orange).

Evolocumab administration resulted in significant reductions in median LDL cholesterol and Lp(a) levels (121 mg/dL to 64 mg/dL, −50.0%, p < 0.001; 51.8 nmol/L to 39.1 nmol/L, −23.6%, p = 0.002, respectively, Fig. 1A and B). In contrast, the median plasma CoQ10 concentration remained unchanged (1028.5 nmol/L to 1002.5 nmol/L, −0.2%, p = 0.33, Fig. 1C).

### 3.3 Correlation between changes in serum lipids and CoQ10

The percent change in LDL cholesterol showed no correlation with the percent change in Lp(a) (Y = −0.011X − 23.21, R^2^ = 0.00002, Supplemental Fig. 1A). Similarly, the percent change in CoQ10 was not correlated with changes in LDL cholesterol (Y = 0.0836X + 2.8838, R^2^ = 0.0091, Supplemental Fig. 1B) or Lp(a) (Y = 0.1045X + 1.0019, R^2^ = 0.00736, Supplemental Fig. 1C).

## 4 Discussion

In this study, we examined serum lipid profiles and plasma CoQ10 levels before and after the addition of evolocumab in patients with HeFH who were receiving rosuvastatin and ezetimibe, and assessed the associations between CoQ10 changes and reductions in LDL cholesterol and Lp(a). We found that evolocumab did not decrease plasma CoQ10 concentrations, despite producing significant reductions in LDL cholesterol and Lp(a).

Statins are essential therapeutic agents for the prevention of atherosclerotic cardiovascular disease and are particularly indispensable for patients with FH, who exhibit markedly elevated LDL cholesterol levels from birth. However, a subset of patients develop muscle-related symptoms during statin therapy, a condition referred to as statin intolerance. Although the mechanisms underlying statin intolerance remain incompletely understood, one proposed explanation is that inhibition of HMG-CoA reductase reduces hepatic synthesis of CoQ10, leading to decreased circulating levels [8,9]. Indeed, CoQ10 supplementation has been reported to alleviate symptoms in some patients with statin intolerance [10].

In clinical practice, PCSK9 inhibitors are frequently used in combination with statins and ezetimibe in patients with FH. However, the effects of PCSK9 inhibitors on circulating CoQ10 levels have not been previously clarified. Although PCSK9 inhibitors and statins both reduce LDL cholesterol via upregulation of LDL receptors, their muscle-related adverse effect profiles differ substantially. Our findings provide several insights. First, the discrepancy in muscle symptoms between statins and PCSK9 inhibitors may be attributable to their differing effects on circulating CoQ10. Second, plasma CoQ10 levels do not appear to be influenced by LDL receptor activation itself. Third, CoQ10 concentrations are not associated with LDL cholesterol or Lp(a) levels. Collectively, these observations suggest that for patients experiencing statin-associated muscle symptoms, clinically feasible strategies include CoQ10 supplementation or the use of PCSK9 inhibitors. However, we have to acknowledge the exploratory nature of this study, especially the absence of clinical endpoints (e.g., muscle symptoms, serum creatine kinase levels). Accordingly, large scale confirmatory clinical trials with these assessments would be needed in the future.

This study has several limitations. First, the sample size was small, consisting of only 10 patients with HeFH. However, the uniformity of treatment—since all patients received the same statin and ezetimibe regimen—may have reduced potential confounding. Second, none of the participants were truly statin-intolerant, and therefore extrapolation of these findings to statin-intolerant populations should be made with caution. Third, the study was conducted exclusively in Japanese patients, limiting the generalizability of the results to other ethnic groups. Fourth, CoQ10 levels in this study were not normalized to LDL cholesterol nor total cholesterol levels. However, we believe that this may not affect the results so much, since their LDL cholesterol levels were lowered via medications.

In conclusion, evolocumab did not affect plasma CoQ10 levels, despite significantly lowering LDL cholesterol and Lp(a). This may represent a clinical advantage, as evolocumab can reduce LDL cholesterol without contributing to muscle symptoms, even in patients who are intolerant to statins.

## Ethical approval

Ethics Committees of Kanazawa University (No. 1965).

## CRediT authorship contribution statement

Study conception: HT, YT and MT; data collection: HT, YT and MT. Analysis and interpretation of results: HT, YT and MT; draft manuscript preparation: HT, YT and MT. All authors reviewed the results and approved the final version of the manuscript.

## Declaration of generative AI in scientific writing

None.

## Funding

This work has been supported by a grant from the 10.13039/100009647Ministry of Health, Labor and Welfare of Japan (Sciences Research Grant for Research on Rare and Intractable Diseases), and a grant from 10.13039/100009619Japan Agency for Medical Research and Development (AMED: 25130864).

## Declaration of competing interest

The authors declare that they have no known competing financial interests or personal relationships that could have appeared to influence the work reported in this paper.
