# Supplementary material for: Effects of evolocumab on plasma coenzyme Q10 in patients with heterozygous familial hypercholesterolemia
Source: Atheroscler Plus. 2026 Jun 19;65:100575. doi: 10.1016/j.athplu.2026.100575 (PMC13320416; doi:10.1016/j.athplu.2026.100575)
Supplement: Multimedia component 1 [file mmc1.pptx]

## Slide 1
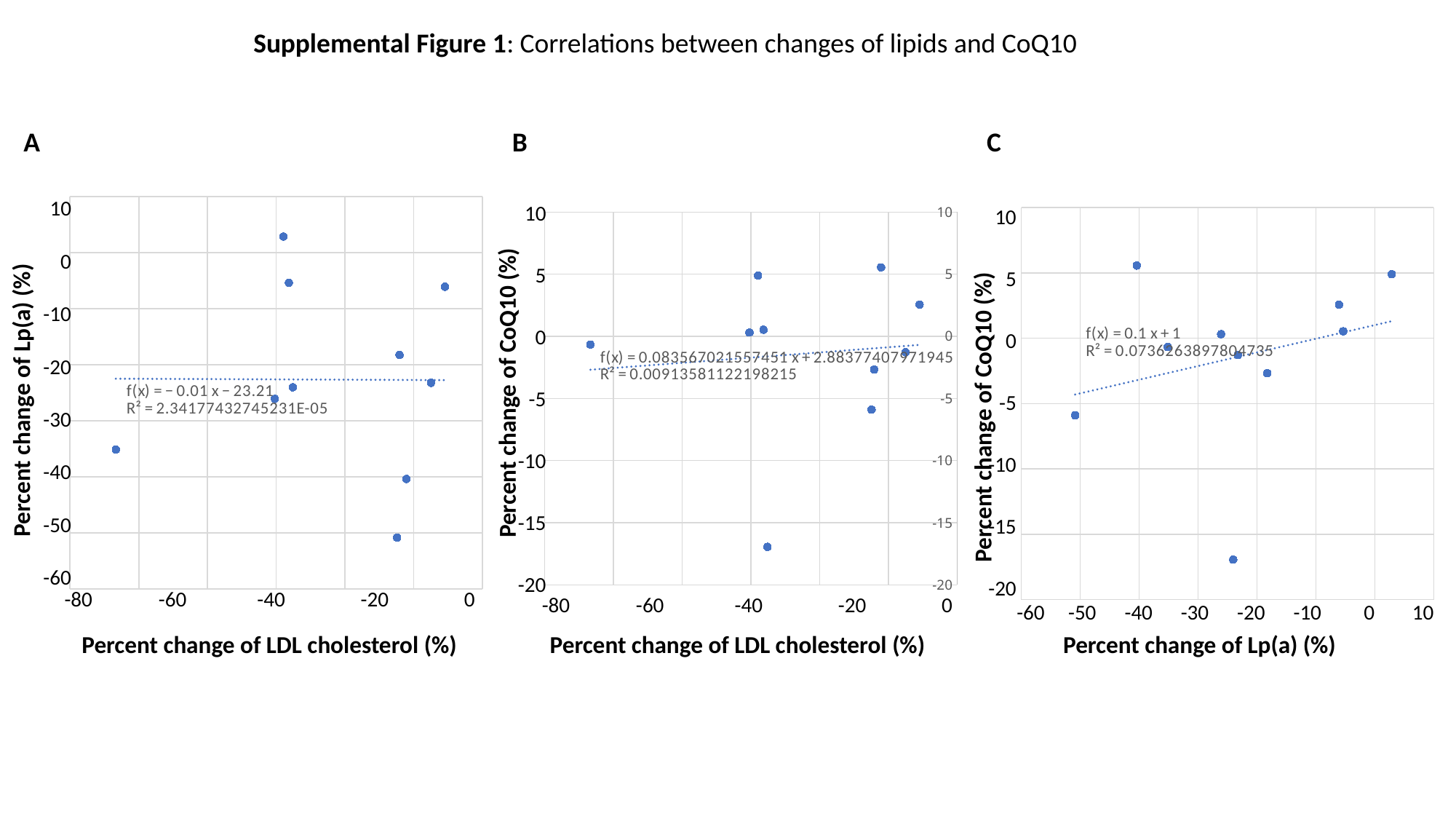

Supplemental Figure 1: Correlations between changes of lipids and CoQ10
A
B
C
### Chart
| Category | |
|---|---|10
0
-10
-20
-30
-40
-50
-60
### Chart
| Category | |
|---|---|10
5
0
-5
-10
-15
-20
10
5
0
-5
-10
-15
-20
### Chart
| Category | |
|---|---|Percent change of CoQ10 (%)
Percent change of Lp(a) (%)
Percent change of CoQ10 (%)
-80 -60 -40 -20 0
-80 -60 -40 -20 0
-60 -50 -40 -30 -20 -10 0 10
Percent change of LDL cholesterol (%)
Percent change of LDL cholesterol (%)
Percent change of Lp(a) (%)
